# Perceptual Fluency Affects Judgments of Learning Non-analytically and Analytically Through Beliefs About How Perceptual Fluency Affects Memory

**DOI:** 10.3389/fpsyg.2020.552824

**Published:** 2020-10-02

**Authors:** Zhiwei Wang, Chunliang Yang, Wenbo Zhao, Yingjie Jiang

**Affiliations:** ^1^School of Psychology, Northeast Normal University, Changchun, China; ^2^Institute of Developmental Psychology, Faculty of Psychology, Beijing Normal University, Beijing, China

**Keywords:** judgments of learning, perceptual fluency, beliefs, font size effect, metamemory, metacognition

## Abstract

Perceptual fluency is generally thought to affect judgments of learning (JOLs) non-analytically. However, some studies suggested that perceptual fluency may also affect JOLs analytically based on beliefs about the relationship between perceptual fluency and memory performance. The present study aimed to investigate how perceptual fluency affects JOLs. In Experiment 1, participants performed a continuous identification task and a JOLs task to determine whether perceptual fluency affects JOLs. In Experiment 2, we manipulated participants’ beliefs about how perceptual fluency affects memory to explore whether perceptual fluency affects JOLs through belief-based analysis. In Experiment 3, we explored whether participants who believed neither perceptual fluency nor font size affected memory performance still offered higher JOLs to large words than to small words, to explore whether perceptual fluency affects JOLs non-analytically. In Experiment 4, participants performed a continuous identification-JOLs task, and then they performed an observation task to measure their beliefs about fluency and memory. The results of the four experiments suggested that perceptual fluency affects JOLs both non-analytically and analytically based on beliefs about the relationship between perceptual fluency and memory performance.

## Introduction

Judgments of learning (JOLs) refers to predictions of the likelihood of correctly retrieving studied materials in a subsequent memory test (Metcalfe, 2009). It is an important index of metamemory monitoring (Metcalfe, 2002).

As for the mechanism of JOLs, the most widely accepted theory is the cue-utilization framework proposed by Koriat (1997). According to the cue-utilization framework, JOLs are inference based on some available cues when people are making JOLs (Susser and Mulligan, 2015; Susser et al., 2017; Undorf et al., 2018, 2020; Undorf and Bröder, 2020). JOLs are based on three different types of cues: intrinsic cues, extrinsic cues, and mnemonic cues.

Processing fluency is one of the most critical mnemonic cues (Begg et al., 1989; Koriat, 1997; Bjork, 1999; Koriat and Levy-Sadot, 1999). Koriat et al. (2004, p. 653) even argued that “JOLs are based predominantly—perhaps exclusively—on the subjective experience associated with processing fluency.” The mnemonic cues affect JOLs in an implicit, non-analytical way (Koriat, 1997; Koriat and Levy-Sadot, 1999; Koriat et al., 2004). Rhodes and Castel (2008) manipulated perceptual fluency (a kind of processing fluency) with font size to investigate the effect of perceptual fluency on JOLs. The results showed that participants gave higher JOLs to large words than to small words, but at the same time, font size did not affect memory performance. Even when participants were explicitly told that font size did not affect memory performance, they still offered large words higher JOLs. However, when the fluency difference was eliminated by presenting words in an alternating format (e.g., *PiAnO*), the font size effect disappeared. The results of the above series of experiments support the opinion that perceptual fluency as a mnemonic cue affects JOLs in a non-analytical way.

However, some research results suggested that perceptual fluency may also affect JOLs in an analytical way. Miele et al. (2011) found that the effect of font size on JOLs was moderated by beliefs about intelligence. Participants who viewed intelligence as fixed offered higher JOLs to large words than to small words, while participants who viewed intelligence as malleable gave the same JOLs to large and small words. The results suggested that perceptual fluency may affect JOLs through belief-based analysis.

Yang et al. (2018) aimed to explore whether beliefs about fluency mediate the effect of perceptual fluency on JOLs. In their Experiment 3, participants first performed a continuous identification-JOLs task, in which they identified words in either large or small font sizes, and made item-by-item JOLs (sJOLs; JOLs made in the study task). In addition, they were asked to perform an observation task, in which they were instructed to watch the learning process of another participant (actually from himself/herself) and make JOLs (oJOLs; JOLs made in the observation task) to predict the likelihood that another participant would recall correctly. In the observation task, instead of watching the real words, the participants only saw meaningless letter strings (i.e., *abcde*) presented in the same font size and for the same duration as the item in the continuous identification-JOLs task. Because in the observation task the participants could not experience the learning process, they could only make JOLs through beliefs about the relationship between fluency and memory. Yang et al. conducted a multilevel mediation analysis to explore whether beliefs about fluency and memory mediate the effect of fluency on JOLs. However, the results showed that the indirect effect of perceptual fluency (RTs) on JOLs through beliefs (oJOLs) was not significant. The results did not support the claim that fluency affects JOLs through beliefs about fluency and memory.

However, there are two possible reasons for this null result. First, beliefs about fluency are not a mediating variable but a moderating variable. However, they analyzed the data in a mediating framework. Second, using oJOLs as an indicator of belief about fluency may be inappropriate. In the Experiment 3 of Yang et al. (2018), the font size of items in the observation task was the same as that in the study task. Under such a condition, the oJOLs not only reflected the beliefs about the relationship between perceptual fluency and memory performance but also the beliefs about the relationship between font size and memory performance. The results of their analysis showed that oJOLs were affected by the font size but not by the RTs. So, beliefs reflected by oJOLs were more likely to be “the large items will get better memory performance” than “the item with higher perceptual fluency will get better memory performance.” If so, it is no surprise that the indirect effect of perceptual fluency (RTs) on JOLs through beliefs (oJOLs) was not significant. Similarly, Experiment 2 of Chen et al. (2019) tried to examine whether the effect of font size on JOL was moderated by beliefs about perceptual fluency and memory. They manipulated participants’ beliefs about the relationship between perceptual fluency and memory and found that participants who believe high perceptual fluency words are easy to remember offered higher JOLs to large words than to small words, while participants who believe perceptual fluency does not affect memory did not offer higher JOLs to large words than to small words. But they manipulate beliefs using the instruction: “Large words are easy to process than small words. The Ease of processing will reduce the cognitive load and help memory, so large words are easy to remember than small words (P158).” In this case, the participants’ beliefs still contain beliefs about fluency and memory and beliefs about font size and memory. So, it cannot be concluded that the perceptual fluency can affect the JOLs in an analytical way based on beliefs about fluency and memory.

If perceptual fluency can affect JOLs through beliefs about fluency and memory analytically, then another problem arose: whether perceptual fluency affects JOLs non-analytically. The previous researchers generally hold the affirmative opinion (Koriat, 1997; Koriat et al., 2004; Koriat and Bjork, 2006; Yang et al., 2018). However, some researchers take a different view. Susser et al. (2016) explored this question by employing an identity-priming paradigm. They asked participants to name and then make item-by-item JOLs for each word, which was preceded by a matched or mismatched prime. They found that regardless of whether the primes were obvious or not, naming latencies were shorter for matched items than mismatched items, while JOLs were higher for matched items than mismatched items only when primes are obvious. Susser et al. came to the conclusion that perceptual fluency influenced JOLs only when belief-based information was available. However, here is another possibility that the difference of perceptual fluency in inconspicuous conditions may be not big enough to make a significant impact on JOLs. Therefore, so far, it has not been concluded that perceptual fluency, *per se*, cannot influence JOLs in a non-analytical way. So, the present study aimed to investigate how perceptual fluency affects JOLs: in an analytical way, in a non-analytical way, or in both ways.

However, to our knowledge, whether perceptual fluency affects JOLs remains controversial. Studies of Mueller et al. (2014), Susser et al. (2016), and Su et al. (2018) did not support the claim that perceptual fluency affects JOLs. However, studies of Besken and Mulligan (2014), Besken (2016), Undorf et al. (2017), and Yang et al. (2018) support this claim. Moreover, in study of Yang et al. (2018), the continuous identification task was followed immediately by the JOLs task. The continuous identification task may make perceptual fluency more pronounced than in the absence of continuous identification tasks. This may make the participants more dependent on perceptual fluency when making JOLs. So, in Experiment 1, we repeated Experiment 1 of Yang et al. (2018), but the continuous identification task and JOLs task were separated, to reduce the impact of the continuous identification task on JOLs. In Experiment 2, we manipulated the participants’ beliefs about the relationship between perceptual fluency and memory performance to explore whether perceptual fluency affects JOLs through beliefs about fluency and memory. In Experiment 3, we explored whether participants who believed that memory performance had nothing to do with perceptual fluency and font size gave higher JOLs to large words than to small words, to confirm whether perceptual fluency, *per se*, affects JOLs in a non-analytical way. In our Experiment 2 and Experiment 3, we did not measure perceptual fluency. So, in Experiment 4, we asked the participants to complete a continuous identification-JOLs task to measure perceptual fluency, and then we asked them to perform an observation task to measure their beliefs about fluency and memory. In the observation task, all words were shown in 32-pt to make sure that the oJOLs reflect only beliefs about the relationship between perceptual fluency and memory, and not beliefs about the relationship between font size and memory.

## Experiment 1

### Participants

We conducted a power analysis using G*power 3.1 to determine the required sample size. The effect sizes (Cohen’s *ds*) from previous studies using Chinese words as experimental materials and presenting the words with 9-pt and 70-pt fonts ranged from 0.58 to 1.13 (Hu et al., 2015; Su et al., 2018). By using the effect size 0.58–1.13, we found that 9–27 participants are required to observe a significant (*α* = 0.05) font size effect on JOLs at 0.90 power. We recruited 28 undergraduates (20 females) with a mean age of 20.11 (*SD* = 2.30) years from Northeast Normal University. All participants had a normal or corrected-to-normal vision, and their first language was Chinese. Each participant was tested individually and received 20 RMB as a reward after the experiment. Written informed consent was obtained from participants in all experiments of this study. All the experiments in this study were approved by The Ethics Committee of Northeast Normal University.

### Materials and Apparatus

The materials consisted of 64 Chinese words (eight of them are for practice). The logarithm (base 10) of the numbers of occurrences of these words in CCL corpus was between 2.34 and 4.71 (*M* = 3.53, *SD* = 0.56; Zhan et al., 2019). As in previous studies using Chinese words as materials, one set of words was presented in 9-pt font, and the other set was presented in 70-pt font (Hu et al., 2015, 2016; Su et al., 2018). Which set of words were presented in 9-pt (70-pt) was counterbalanced between participants. The two sets of words did not differ in the word frequency or the number of strokes (*p*s > 0.05). The experiment was conducted with E-prime2.0. Stimuli were displayed on 24-inch monitors with a refresh rate of 85 Hz and a resolution of 1,024 × 768.

### Procedure

The procedure was similar to Experiment 1 of Yang et al. (2018), except that the continuous identification task and JOLs task were separated. The procedure consisted of four phases: the continuous identification task phase, the JOLs task phase, the distractor task phase, and the memory test phase. In the continuous identification task phase, each trial started with the presentation of a black cross in medium font size (30-pt) at the center of the screen for 500 ms. Then a word (9-pt/70-pt) and a mask (10-pt/74-pt) were alternately presented in Courier New. The extremely rare (the number of occurrences in CCL corpus was 0) Chinese character “鳠鳠” was used as the mask. Before the experiment, participants were asked if they know the word. No one reported knowing the word. The masks were presented a little larger than the target words to ensure that the target words were completely masked. There were 14 cycles in each trial. In the first cycle, a word was first presented for 17 ms and replaced by the masking stimulus presented for 238 ms. Then the second cycle began. The presentation duration of the word in the N cycle was equal to the presentation duration of the N-1 cycle plus 17 ms, while the presentation duration of the masking stimulus in the N cycle was equal to the presentation duration of the N-1 cycle minus 17 ms. Participants were asked to focus on the stimulus on the screen and press the ENTER key as soon as they recognized the word. When the participants made a response or the total of 14 cycles were over, the next trial began. If they responded, the word and mask disappeared, and participants were asked to say the words aloud, and the experimenter recorded them with another computer. Words were presented in random order.

In the JOLs phase, the same 56 words were presented one by one for 2 s in a renewed random order. Immediately following the presentation of each word, a slider ranging from 0 to 100 appeared in the center of the monitor. Zero denoted “I am pretty sure I will not be able to recall this word” and 100 denoted “I am pretty sure I will be able to recall this word.” Participants were instructed to predict the likelihood that the word would be correctly recalled in a subsequent memory test by clicking on the corresponding position on the slider. After the JOLs phase, learners engaged in a distractor task (e.g., 67 + 23 = ___?) for 90 s. Then they took a memory test, in which they were instructed to write as many words as they could on a blank sheet of paper, not needed to be in order. There was no time limit for the recall test. When the recall time was longer than 5 min, the participants were reminded to finish if she/he fell it was too difficult to recall more. If they thought they could recall more, they could continue to recall.

### Results and Discussion

All data in this study were analyzed using RStudio. If not explicitly stated, Rstudio’s own package is used. The identification accuracy, identification RTs, JOLs, and Recall accuracy are shown in Table 1. Since the identification accuracy of the participants did not follow a normal distribution, the Wilcoxon signed rank test was used to compare the identification accuracy of 9-pt and 70-pt words. The result showed that there was no difference in identification accuracy between large (*M* = 97.06%, *SD* = 4.13%) and small words (*M* = 96.17%, *SD* = 5.23%), *p* = 0.67, 95% CI (Cumming, 2012) = [−3.58%, 5.36%]. In the following analysis, all data from incorrectly identified trials were excluded.

**Table 1:** *M* (*SD*) of participants’ identification accuracy, identification RTs, judgments of learning (JOLs), and recall accuracy in Experiments 1–4.

	Identification accuracy	Identification RTs	JOLs	Recall accuracy
**Experiment 1**			
9-pt	96.17 (5.23)	1.75 (0.35)	51.24 (14.35)	28.50 (7.56)
70-pt	97.06 (4.13)	1.43 (0.32)	64.40 (14.11)	27.89 (8.09)
**Experiment 2**			
**Group 1**			
9-pt			46.55 (18.77)	30.00 (10.90)
70-pt			57.24 (18.12)	31.88 (14.55)
**Group 2**			
9-pt			56.18 (15.42)	33.13 (14.52)
70-pt			55.56 (16.93)	32.00 (13.66)
**Experiment 3**			
9-pt			43.60 (16.20)	27.51 (10.42)
70-pt			47.83 (17.80)	28.78 (7.36)
**Experiment 4**			
**Study task**			
9-pt	94.55 (6.76)	1.61 (0.31)	47.09 (17.26)	28.32 (17.89)
70-pt	95.23 (4.87)	1.37 (0.35)	52.00 (16.95)	26.33 (17.74)
**Observation task**			
9-pt			50.49 (15.70)	
70-pt			50.81 (15.12)	

**Figure 1:**
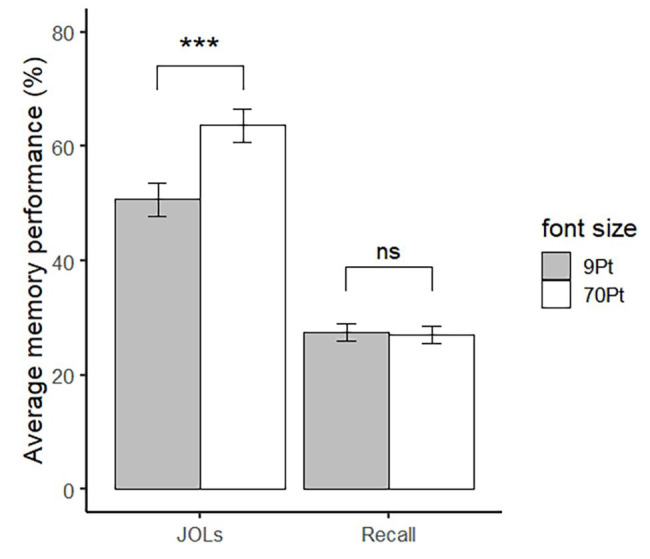
JOLs and recall for large and small words in Experiment 1. Error bar represents ±1 standard error. ^∗∗∗^*p* < 0.001.

Predicted and actual recall performance (recall accuracy = number of words correctly recalled/number of words correctly identified) are presented in Figure 1. There was no difference in recall accuracy between large (*M* = 27.89%, *SD* = 8.09%) and small words (*M* = 28.50%, *SD* = 7.56%), *t*(27) = 0.39, *p* = 0.70 (see the right pair of bars in Figure 1). In contrast, the font size effect appeared. The JOLs of large words (*M* = 64.40, *SD* = 14.11) were significantly higher than small words (*M* = 51.24, *SD* = 14.35), *t*(27) = 5.82, *p* < 0.001, Cohen’s *d* = 1.10 (see the left pair of bars in Figure 1).

Participants’ median identification RTs were significantly shorter for large (*M* = 1.43, *SD* = 0.32) than for small words (*M* = 1.75, *SD* = 0.35), *t*(27) = 6.64, *p* < 0.001, Cohen’s *d* = 1.25. This result indicated that the font size of the words affected perceptual fluency.

To explore whether the font size effect on JOLs was mediated by fluency (RTs), a multilevel mediation analysis was conducted with the R bmlm package, which provides a Bayesian estimation of multilevel mediation models (Vuorre, 2017). In this analysis, we took font size (small = 0; large = 1) as the independent variable, RTs as the mediator, and JOLs as the dependent variable. The mediation effect was estimated with four Markov Chain Monte Carlo (MCMC) chains and 10,000 iterations for each chain. The results are shown in Table 2. The total effect of font size on JOLs was significant, *b* = 13.20, 95% CI = [8.31, 17.96]. The indirect effect of font size on JOLs through RTs was significant, *b* = 0.82, 95% CI = [0.05, 1.62], indicating that large fonts increase JOLs indirectly by increasing perceptual fluency. Fluency (RTs) explained 6%, 95% CI = [0%, 14%], of the font size effect on JOLs. The direct effect of font size on JOLs was still significant when RTs were controlled, *b* = 12.38, 95% CI = [7.54, 17.12].

**Table 2:** Multilevel mediation analysis results in Experiment 1.

	*b*	*SE*	95% CI
Effect of font size on RTs	−0.37	0.04	[−0.46, −0.29]
Effect of RTs on JOLs	−2.30	0.95	[−4.18, −0.37]
Total effect of font size on JOLs	13.20	2.45	[8.31, 17.96]
Direct effect of font size on JOLs	12.38	2.45	[7.54, 17.12]
Indirect effect of font size on JOLs through RTs	0.82	0.40	[0.05, 1.62]
Proportion of the total effect of font size on JOLs mediated by RTs	6%	3%	[0%, 14%]

In Experiment 1, we tried to repeat Experiment 1 of Yang et al. (2018) using Chinese words as experimental materials, when the continuous identification task and JOLs task were separated. In Yang et al.’s study, the continuous identification task was followed immediately by the JOLs task. The continuous identification task may make perceptual fluency more pronounced than in the absence of continuous identification tasks and may lead to an overestimation of the effect of perceptual fluency on JOLs. In Yang et al.’s study, perceptual fluency explained 21% in Experiment 1 and 15% in Experiment 3 of the font size effect on JOLs. However, in our Experiment 1, perceptual fluency (RTs) explained only 6% of the font size effect on JOLs. In Experiment 1, the continuous identification task and JOLs task were separated, so the experienced fluency in the continuous identification task and JOLs task may not exactly correspond, which may lead to an underestimation of the effect of perceptual fluency on JOLs. Anyway, although the proportion of perceptual fluency explaining the font size effect was numerically smaller than that in the study of Yang et al. (2018), the most important thing was that the result of Experiment 1 supported the claim that perceptual fluency manipulated by font size affects JOLs (Besken and Mulligan, 2014; Besken, 2016; Undorf et al., 2017; Yang et al., 2018). This provided the basis for the follow-up experiments of how perceptual fluency affects JOLs.

## Experiment 2

The result of Experiment 1 showed that perceptual fluency affect JOLs. In Experiment 2, we manipulated participants’ beliefs about the relationship between perceptual fluency and memory performance. If perceptual fluency can affect JOLs through belief-based analysis, the font size effect on JOLs will be moderated by beliefs about perceptual fluency and memory. If perceptual fluency cannot affect JOLs through belief-based analysis, the font size effect on JOLs will not be moderated by beliefs about perceptual fluency and memory.

### Participants

As in Experiment 1, by using the effect size 0.58–1.13, we found that a total of 24–42 participants are required to observe a significant (*α* = 0.05) effect at 0.90 power. In order to get enough qualified participants, we recruited 69 undergraduates (51 females) with a mean age of 20.36 (*SD* = 2.57) years from Northeast Normal University and Jilin Medical University, of which 34 were in the belief 1 group and 35 were in the belief 2 group. All participants had a normal or corrected-to-normal vision, and their first language was Chinese. Each participant was tested individually and received 15 RMB as a reward after the experiment.

### Materials and Apparatus

A set of new words was chosen for Experiment 2. The materials consisted of 72 Chinese words (eight of them are for practice). The logarithm (base 10) of the number of occurrences of these words in CCL corpus was between 2.24 and 4.47 (*M* = 3.39, *SD* = 0.52; Zhan et al., 2019). The number of strokes of the words was between 8 and 22 (*M* = 16.20, *SD* = 3.28). The 72 words were randomly divided into two sets (each contained 36 words). One set of words was presented in 9-pt font, and the other set was presented in 70-pt font. Which set of words were presented in 9-pt (70-pt) was counterbalanced between participants. The two sets of words did not differ in the word frequency or the number of strokes (*p*s > 0.05). The experiment was conducted with E-prime2.0. Stimuli were displayed on 24-inch monitors with a refresh rate of 60 Hz and a resolution of 1,024 × 768.

### Procedure

After providing informed consent, participants were given the information that perceptual fluency is the subjective experience of the easy of stimulus processing caused by the perceptual characteristics of the stimulus. Then participants in the belief 1 group (instruct participants to believe that words with high perceptual fluency will be remembered better) were given the following information:

*Psychological research has shown that words with high perceptual fluency will be remembered better because processing fluent information needs less effort than disfluent information*.

Participants in the belief 2 group (instruct participants to believe that words with low perceptual fluency will be remembered better) were given the following information:

*Psychological research has shown that words with low perceptual fluency will be remembered better because disfluent information leads to stronger physiological arousal and make people put in more effort*.

After receiving the instructions, participants need to report whether they fully understood the instructions. If they fully understood the instructions, they completed a study-JOLs task. In this task, learners were required to study 64 words with a 5s presentation time for each word. Immediately following the presentation of each word, learners were instructed to type a number from 0 to 100 that represented the probability of recalling that word in a memory test about 10 min later. Words were presented in random order. After completing the study-JOLs task, participants were asked: “Which words are better remembered, perceptual fluent words or perceptual disfluent words, or is it just as good?” Then they completed a 90s distractor task and memory test as in Experiment 1.

### Results and Discussion

Among the 69 participants, two participants gave each word the same JOLs, so the data of these two participants were excluded. We first analyzed the results of the investigation after the study-JOLs phase. In the belief 1 group, 25 participants believed that items with higher perceptual fluency would get better memory performance. Similarly, in the belief 2 group, 25 participants believed that items with higher perceptual fluency would get lower memory performance. So we analyzed the data only including the participants who still believed the experiment instructions after the study-JOLs phase.

For recall performance, a mixed-design ANOVA was conducted with font size (9-pt vs. 70-pt) as the within-subject factor and belief (belief 1 vs. belief 2) as the between-subject factor. The main effect of font size, *F*(1,48) = 0.03, *p* = 0.858, the main effect of belief, *F*(1,48) = 0.26, *p* = 0.613, and the interaction between font size and belief, *F*(1,48) = 0.52, *p* = 0.476, were all insignificant.

**Figure 2:**
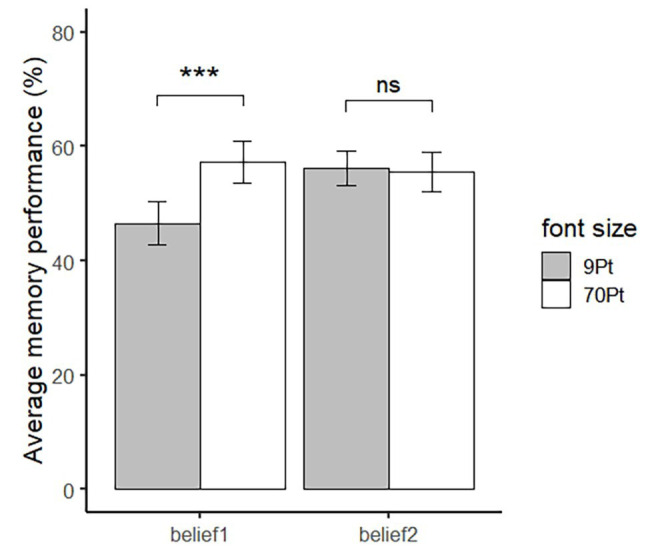
JOLs for large and small words of participants in different belief groups in Experiment 2. Error bar represents ±1 standard error. ^∗∗∗^*p* < 0.001.

For JOLs, a mixed-design ANOVA was conducted with font size (9-pt vs. 70-pt) as the within-subject factor and belief (belief 1 vs. belief 2) as the between-subject factor. The interaction between font size and belief was significant, *F*(1,48) = 12.97, *p* < 0.001, partial *η*^2^ = 0.21. A further simple effect analysis showed that the JOLs of large words (*M* = 57.24%, *SD* = 18.12%) were significantly higher than small words (*M* = 46.55%, *SD* = 18.77%) in the belief 1 group, *F*(1,24) = 23.17, *p* < 0.001 (see the left pair of bars in Figure 2), while there was no difference in JOLs between large (*M* = 55.56%, *SD* = 16.93%) and small words (*M* = 56.18%, *SD* = 15.42%) in the belief 2 group, *F*(1,24) = 0.08, *p* = 0.782 (see the right pair of bars in Figure 2).

#### Does Perceptual Fluency Affect Judgments of Learning Analytically?

We further calculated the effect size (Cohen’s *d*) of the font size on JOLs of each participant. Next, we compared the effect size of the belief 1 group and the belief 2 group. The result showed that the effect size was larger in the belief 1 group (*M* = 0.58, *SD* = 0.44) than that in the belief 2 group (*M* = 0.05, *SD* = 0.44), *t*(48) = 4.25, *p* < 0.001, Cohen’s *d* = 1.04. The results showed that the effect of font size on JOLs was moderated by beliefs about the relationship between perceptual fluency and memory.

For participants who believed that the memory performance of fluent words would be better than disfluent words, the effect of font size on JOLs appeared. In contrast, for participants who believed that memory performance of disfluent words will be better than fluent words, there was no difference in JOLs between words in different font sizes. The result that changing the beliefs about the relationship between perceptual fluency and memory performance, in turn, changed the font size effect suggested that fluency affect JOLs in an analytical way based on beliefs about how perceptual fluency affects memory.

However, perceptual fluency may only partially affect JOLs through beliefs. If perceptual fluency affects JOLs completely through beliefs, participants who believed that the memory performance of disfluent words was better than fluent words should show a reversed font size effect. However, there was neither a font size effect nor a reversed font size effect. The null result suggested that perceptual fluency may also affect JOLs in a non-analytical way and counteract with the fluency belief that items with higher perceptual fluency will get lower memory performance.

However, another possibility for the null result in the belief 2 group was that participants may hold other beliefs about the relationship between font size and memory performance, such as “the large items are more distinctive, and more distinctive items will get better memory performance” and “the large items are more important, and more important items will get better memory performance” (Kornell et al., 2011; Mueller et al., 2014; Kornell and Hausman, 2017). These beliefs about font size and memory performance may counteract with the fluency belief that items with higher perceptual fluency will get worse memory performance. So in Experiment 3, we explored whether perceptual fluency affects JOLs in a non-analytical way by checking whether participants who believed memory performance had nothing to do with perceptual fluency and font size still offer higher JOLs to large words than to small words.

## Experiment 3

### Participants

In Experiment 3, we induced participants to believe that memory performance has nothing to do with perceptual fluency and font size. The effect of font size on JOLs may be diminished in such condition, so we recruited more participants than those in Experiment 1. We recruited 71 undergraduates (55 females) with a mean age of 20.60 (*SD* = 2.23) years from Northeast Normal University and Jilin Medical University. All participants had a normal or corrected-to-normal vision, and their first language was Chinese. Each participant was tested individually and received 15 RMB as a reward after the experiment.

### Materials and Apparatus

The materials and apparatus are the same as those used in Experiment 2.

### Procedure

After providing informed consent, participants were given the information that perceptual fluency is the subjective experience of the easy processing caused by the perceptual characteristics of the stimulus. Then they were told that neither perceptual fluency nor font size of the words affects memory performance. The guidance materials are as follows:

Memory is affected by the difficulty of semantic processing, but not affected by the difficulty of perceptual processing. The researchers manipulated the difficulty of semantic processing of word pairs by the semantic association of the two words and manipulated the difficulty of perceptual processing by font size. Results showed that the word pairs with higher semantic association had better memory performance, which was consistent with the participants' expectations. However, although participants believed that the large words, whose perceptual fluency was higher, would be remembered better, in actual, there was no difference in the memory performance between large and small words.

After receiving the instructions, participants need to report whether they fully understood the instructions. If they fully understood the instructions, they completed the study-JOLs task as in Experiment 2. After completing the study-JOLs task, we administered a questionnaire to each participant. They were asked two questions: “Which words are better remembered, perceptual fluent words or perceptual disfluent words, or is it just as good?” and “Which words are better remembered, the large words or the small words, or is it just as good?” Then they completed a 90s distractor task and memory test as in Experiment 2.

### Results and Discussion

Among the 71 participants, four participants gave each word the same JOLs, so the data of these four participants were excluded. We first analyzed the questionnaire after the study-JOLs phase. Only 37 participants reported they believed neither perceptual fluency nor font size of the words affect memory performance. So the data we analyzed included only the above 37 participants.

**Figure 3:**
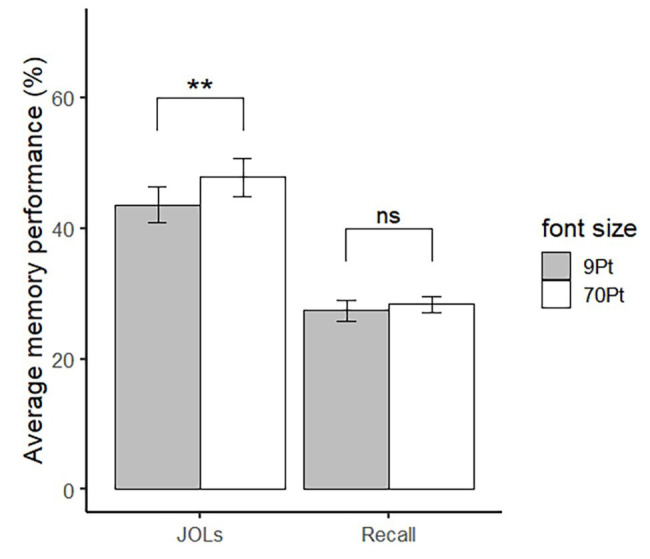
JOLs and recall for large and small words of qualified participants in Experiment 3. Error bar represents ±1 standard error. ^∗∗^*p* < 0.01.

Consistent with previous experiment, there was no difference in recall accuracy between large (*M* = 28.78%, *SD* = 7.36%) and small words (*M* = 27.51%, *SD* = 10.42%), *t*(36) = 0.78, *p* = 0.44 (see the right pair of bars in Figure 3). In contrast, the font size effect still existed. The JOLs of large words (*M* = 47.83, *SD* = 17.80) were significantly higher than small words (*M* = 43.60, *SD* = 16.20), *t*(36) = 3.11, *p* = 0.004, Cohen’s *d* = 0.51 (see the left pair of bars in Figure 3).

Experiment 3 showed that even if participants believed memory performance had nothing to do with perceptual fluency and font size, they still gave higher JOLs to large words than to small words. In this case, the effect of font size on JOLs cannot be attributed to beliefs about the relationship between perceptual fluency and memory performance or beliefs about the relationship between font size and memory performance. Then we can confirm that perceptual fluency can affect JOLs in a non-analytical way.

## Experiment 4

The results of Experiment 2 suggested that perceptual fluency affected JOLs through beliefs about perceptual fluency and memory in an analytical way, and the results of Experiment 3 suggested that perceptual fluency also affected JOLs in a non-analytical way. However, in Experiment 2 and Experiment 3, we did not measure perceptual fluency directly. So, in Experiment 4, we measured both the perceptual fluency and participants’ beliefs about the relationship between perceptual fluency and memory performance. Besides, manipulating the participants’ beliefs before the JOLs task may make participants more prone to use the belief-based analytical approach in making JOLs. So in Experiment 4, we measured the participants’ beliefs about fluency and memory after the JOLs task.

### Participants

Referring to the number of participants in Experiment 2, we recruited 60 undergraduates (45 females) with a mean age of 19.03 (*SD* = 1.64) years from Northeast Normal University. All participants had a normal or corrected-to-normal vision, and their first language was Chinese. Each participant was tested individually and received 25 RMB as a reward after the experiment.

### Materials and Apparatus

The materials and apparatus are the same as those used in Experiment 2.

### Procedure

Because the design of Experiment 1 caused the effect of perceptual fluency to be underestimated, and we want to compare the result of Experiment 4 with Experiment 3 of Yang et al. (2018), we followed the design of Yang et al. instead of our Experiment 1. The procedure was similar to Experiment 3 of Yang et al., except for the following points: (1) in the study task, words were presented in 9-pt and 70-pt and (2) in the observation task, all words were shown in 32-pt, to make sure that the JOLs in observation task only reflect the beliefs about the relationship between perceptual fluency and memory performance. The specific procedure was as follows: participants first completed a continuous identification-JOLs task (sJOLs; JOLs made in the study task). This task was the similar to Experiment 1, with the main difference that JOLs were made immediately after the stimulus was correctly recognized and typed. Then, they completed an observation task. In the observation task, participants were asked to view another participant’s identification trials and make item-by-item JOLs (oJOLs; JOLs made in the observation task) predicting the other participant’s remembering likelihood. In reality, all participants were shown their own identification trials, but all words were replaced by the same Chinese word “目标(target)” presented in 32-pt for the same duration as the words in the continuous identification task. After the observation task, they completed the same distractor task and memory test as in Experiment 1.

### Results and Discussion

Since the identification accuracy of the participants did not follow a normal distribution, the Wilcoxon signed rank test was used to compare the identification accuracy of 9-pt and 70-pt words. The result showed that there was no difference in identification accuracy between large (*M* = 95.23%, *SD* = 4.87%) and small words (*M* = 94.55%, *SD* = 6.76%), *p* = 0.63, 95% CI = [−3.00%, 1.50%]. In the following analysis, all data from incorrectly identified trials were excluded.

**Figure 4:**
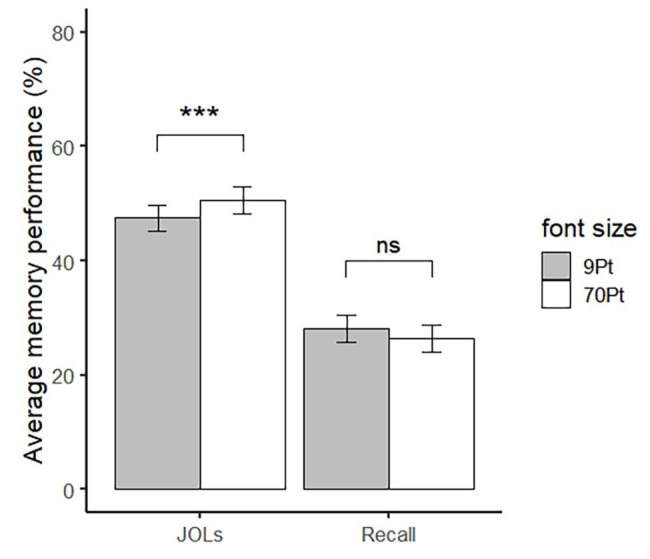
JOLs and recall for large and small words in Experiment 4. Error bar represents ±1 standard error. ^∗∗∗^*p* < 0.001.

The predicted and actual recall performance are presented in Figure 4. Neither recall performance nor the JOLs of the participants fitted a normal distribution, so the Wilcoxon signed rank test was used to compare the differences of memory performance and JOLs between words of different font sizes. Consistent with previous experiments, the result of Wilcoxon signed rank test showed that there was no difference in recall performance between large (*M* = 26.33%, *SD* = 17.74%) and small words (*M* = 28.32%, *SD* = 17.89%), *p* = 0.33, 95% CI = [−2.00%, 5.50%] (see the right pair of bars in Figure 4). In contrast, the result of Wilcoxon signed rank test showed that the JOLs of large words (*M* = 52.00, *SD* = 16.95) were significantly higher than small words (*M* = 47.09, *SD* = 17.26), *p* < 0.001, 95% CI = [−6.19, −2.62] (see the left pair of bars in Figure 4).

Participants’ median identification RTs were significantly shorter for large (*M* = 1.37, *SD* = 0.35) than for small words (*M* = 1.61, *SD* = 0.31), *t*(58) = 7.27, *p* < 0.001, Cohen’s *d* = 0.94. This result indicated that the font size of the words affects the perceptual fluency. The large words were more fluent than small words.

To explore whether the font size effect on JOLs was mediated by fluency (RTs), a multilevel mediation analysis was conducted with the R bmlm package as Experiment 1. The results are shown in Table 3. The total effect of font size on JOLs was significant, *b* = 4.93, 95% CI = [3.43, 6.46]. The indirect effect of font size on JOLs through RTs was significant, *b* = 0.86, 95% CI = [0.31, 1.53], indicating that large fonts increase JOLs indirectly by increasing perceptual fluency. Fluency (RTs) explained 18%, 95% CI = [6%, 31%], of the font size effect on JOLs. The direct effect of font size on JOLs was still significant when RTs were controlled, *b* = 4.07, 95% CI = [2.63, 5.57].

**Table 3:** Multilevel mediation analysis results in Experiment 4.

	*b*	*SE*	95% CI
Effect of font size on RTs	−0.25	0.03	[−0.31, −0.18]
Effect of RTs on JOLs	−1.94	0.80	[−3.52, −0.39]
Total effect of font size on JOLs	4.93	0.77	[3.43, 6.46]
Direct effect of font size on JOLs	4.07	0.75	[2.63, 5.57]
Indirect effect of font size on JOLs through RTs	0.86	0.31	[0.31, 1.53]
Proportion of the total effect of font size on JOLs mediated by RTs	18%	6%	[6%, 31%]

#### Does Perceptual Fluency Affect Judgments of Learning Analytically?

First, we run a regression of sJOLs on RTs for each participant to obtain a slope coefficient *β*_1i_ (an indicator of the effect of fluency on JOLs) and regression of oJOLs on RTs for each participant to obtain a slope coefficient *β*_2i_. If participants held the belief that words with high perceptual fluency would be better remembered, they would give words with short processing time (words with high perceptual fluency) higher oJOLs, then *β*_2i_ was negative, and conversely, *β*_2i_ was positive. So we used the slope coefficient *β*_2i_ of oJOL regression on RT as an index of beliefs about fluency and memory. Then, we run a regression of *β*_1i_ on *β*_2i_, which yield a significant positive slope coefficient, *β* = 0.19, 95% CI = [0.04, 0.33], *p* = 0.011. The result that the effect of perceptual fluency on JOLs was moderated by beliefs of fluency suggested that perceptual fluency affected JOLs in an analytical way.

#### Does Perceptual Fluency Affect Judgments of Learning Non-analytically?

We further analyzed the data from the participants, whose *β*_2_ were greater than 0 (those participants believed that slowly processed items were more likely to be remembered). Twenty-eight participants’ *β*_2_ were greater than 0. For these participants, we first conducted a multilevel regression of oJOLs on RTs with the R lme4 package (Bates et al., 2015) to explore their beliefs about fluency. The fixed effect was 8.26, *p* < 0.001, 95% CI = [5.27, 11.29], indicating that these participants believed that slowly processed items were more likely to be remembered. Next, we conducted a new multilevel regression of sJOLs on RTs to explore the effect of fluency on JOLs among those participants, which yielded a negative slope coefficient, but not reliably, fixed effect was −1.37, *p* = 0.18, 95% CI = [−3.42, 0.59].

The previous analysis showed that perceptual fluency can influence JOLs analytically based on beliefs about the relationship between perceptual fluency and memory performance. However, if perceptual fluency could only influence JOLs through belief-based analysis, those participants who believed that slowly processed items were more likely to be remembered should offer higher JOLs to the disfluent items, but this was not the case. The results suggested that perceptual fluency affected JOLs not only through belief-based analysis but also in a non-analytical way.

## General Discussion

Processing fluency as a mnemonic cue was considered to be an important role in making JOLs (Hertzog et al., 2003; Undorf and Erdfelder, 2011; Besken, 2016; Undorf et al., 2017; Yang et al., 2018). However, so far, it is not clear how perceptual fluency affects JOLs: in an analytical way, in a non-analytical way, or in both ways.

To testify these three hypotheses, we executed four experiments. In Experiment 1, we successfully repeated the Experiment 1 of Yang et al. (2018), when the continuous identification task and JOLs task were separated. The result confirmed that the perceptual fluency influence JOLs. The results of Experiment 1 were consistent with those of Experiment 1 of Yang et al., confirming that the perceptual fluency affect JOLs, which provided the basis for the follow-up experiments of how perceptual fluency affects JOLs.

In Experiment 2, we manipulated participants’ beliefs about the relationship between perceptual fluency and memory performance and found that the font size effect was moderated by the beliefs about fluency and memory. The results of Experiment 2 showed that when participants believed words with high perceptual fluency will be remembered better, they offered higher JOLs to large words than to small words. However, when participants believed words with low perceptual fluency will be remembered better, they did not offer higher JOLs to large words than to small words.

The results are partially consistent with studies about fluency affecting judgments in other domains. Winkielman and Schwarz (2001) asked participants to retrieve 4 (experienced as an easy task) or 12 (experienced as a difficult task) events from their childhood. Part of the participants were told that pleasant memories were difficult to remember, while others were told that unpleasant memories were difficult to remember. The result showed that when the task was difficult (12 events), participants who believed pleasant memories were difficult to remember rated their childhood as happier than participants who believed unpleasant memories were difficult to remember. However, when the task was easy (four events), the pattern was reversed, although not significant. Besides, Briñol et al. (2006) reported similar effects. When ease was described as positive, the attitude of the participants in the easy task was more positive than that of the participants in the difficult task. While when ease was described as negative, the reversed pattern was shown.

Our Experiment 2 found that the effect of fluency was moderated by belief. This is in line with the above studies. However, in their studies, beliefs reversed the direction of the fluency effect. However, in our study, participants who believed words with low perceptual fluency will be remembered better did not show a reserved font size effect. The difference may result from different experimental tasks. The connection between the difficulty of processing materials and memory we have experienced countless times in our daily life. An automatic connection has been established between the two, just like a conditioned reflex. In our Experiment 2, fluency can affect JOLs not only analytically but also automatically. However, the direction of the influence of the two ways was opposite, so there was no reserved effect. However, in the abovementioned studies, participants did not experience the relationship between the fluency of retrieving events from their childhood and whether childhood was happy, and the relationship between the ease of proposing arguments for new examination methods and attitudes toward the new examination method in their daily life. Automatic connection had not been established. Fluency could only work analytically in the above experimental tasks, so there were reversed effects.

Similarly, the results of our Experiment 3 are not consistent with studies about fluency affecting judgments in other domains. In Experiment 3, we explored whether participants who believed memory performance had nothing to do with perceptual fluency and font size still offered higher JOLs to large words than to small words. The result showed that even if participants believed that memory performance had nothing to do with perceptual fluency and font size, they still gave higher JOLs to large words than to small words. However, previous studies showed that when participants became aware that their fluency feelings were not relevant information for evaluating the object, the effects of fluency disappeared or even reversed (for a review, see Unkelbach and Greifeneder, 2013). Oppenheimer (2004) found that when participants were told that fame leads to fluent processing, they did not judge the famous names as frequent names and even judged the famous names as less frequent names. Jacoby and Whitehouse (1989) found that when participants realized that the fluency feelings resulted from whether the context word was matched with the target word or not, they no longer judged more matched target words as old, but instead, they judged fewer matched target words as old.

They interpreted the experiment results with the discounting theory. Discounting is a causal-reasoning phenomenon. If a phenomenon is caused by multiple causes, increasing the confidence in the likelihood of one cause will decrease the confidence in the likelihood of other causes. In the study of Oppenheimer (2004), the role of frequency was underestimated when participants attributed feelings of fluency to fame. Similarly, in the study of Jacoby and Whitehouse (1989), the role of familiarity was underestimated when participants attributed the feelings of fluency to whether the context word was matched with the target word or not.

However, the cognitive processes of predicting memory performance in the present study were not identical to those of the aforementioned studies. In the present study, participants first needed to attribute the fluency experience, that is, to confirm that the perception of fluency was derived from the process of processing the target word. There was no discounting during this process. In the second step, they explain the reason for this fluency experience, that is, the fluency experience results from the characteristics (font size) of the target word itself. There was also no discounting during this process. Then, they need to use the fluency information to predict their future recall performance. This process can be an analytical process or a non-analytical process. When participants believed memory performance had nothing to do with perceptual fluency, perceptual fluency couldn’t influence JOLs analytically. However, perceptual fluency could still affect JOLs in an automatic, non-analytical way. So participants who believed that memory performance had nothing to do with perceptual fluency and font size still offered higher JOLs to large words than to small words.

In Experiment 4, we repeated Experiment 3 of Yang et al. (2018). However, in the observation task, all words were shown in 32-pt to make sure that the JOLs in the observation task only reflect the beliefs about the relationship between fluency and memory. The results confirmed that fluency affects JOLs not only in an analytical way through belief-based analysis but also in a non-analytical way. The results of Experiments 4 and 2 were consistent with Miele et al. (2011). They found that the font size effect was moderated by beliefs about intelligence. However, Miele et al. tested the beliefs about intelligence, but not beliefs about fluency. Based on their research, we examined the effect of beliefs about the relationship between perceptual fluency and memory performance on JOLs. The results confirmed that perceptual fluency affects JOLs through beliefs about fluency and memory in an analytical way.

In Experiments 4 and 3, we found that perceptual fluency can affect JOLs non-analytically, which contradicts the findings of Susser et al. (2016). In their experiments, a matched or mismatched prime word was presented before the target word under subliminal (for 32 ms) or suprathreshold (for 200 ms) condition. They found that perceptual fluency influenced JOLs only when the manipulation of perceptual fluency can be clearly perceived. This may be because under the subthreshold condition, the difference in perceptual fluency caused by whether the prime words matched or not was relatively small, which was not large enough to cause the significant differences in JOLs. In Susser et al.’s Experiment 1, the difference of identification RTs induced by the primes was 35 ms, while in our experiment, the difference of identification RTs induced by the font size was 320 and 310, respectively, in Experiment 1 and Experiment 4, which was almost 10 times that of the former.

### Mechanism of Font Size Effect

**Figure 5:**
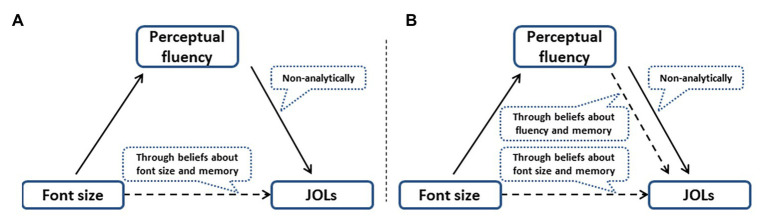
The mechanism of font size effect. Previous studies suggested that there are two main mechanisms for the font size effect (as shown in A). One is belief theory, which believes that people make JOLs analytically through beliefs about font size and memory, and the other one is fluency theory, which believes that people make JOLs non-analytically through subjective feeling about fluency. But according to the results of this study, perceptual fluency can affect JOLs not only in a non-analytical way, but also an analytical way (as shown in B).

Previous studies suggested that there are two main mechanisms for the font size effect (as shown in Figure 5A). The first one is the belief theory, which believes that people hold a priori belief that large words will be remembered better than small words. When people are asked to make JOLs, they will search for cues and beliefs related to this task and reason based on them. In belief theory, font size affects JOLs in an analytical way (Mueller et al., 2014, 2016; Mueller and Dunlosky, 2017). The second one is the fluency theory, which believes that people experience greater fluency when processing large words than processing small words, and the subjective feeling about fluency unconsciously makes people think that large words have been remembered better. In the fluency theory, however, perceptual fluency affects JOLs in a non-analytical way (Koriat and Ma’ayan, 2005; Koriat and Bjork, 2006; Undorf et al., 2017). However, according to the results of this study, perceptual fluency affect JOLs not only in an analytical way through belief-based analysis, but also in a non-analytical way (as shown in Figure 5B).

### How to Manipulate and Measure Perceptual Fluency

In recent years, the influence of perceptual fluency on JOLs has received much attention (Rhodes and Castel, 2008, 2009; Miele et al., 2011; Susser et al., 2013; Besken and Mulligan, 2014; Mueller et al., 2014; Frank and Kuhlmann, 2017; Yang et al., 2018). How to manipulate and measure perceptual fluency has been a key concern. Since perceptual fluency cannot be directly manipulated, it is usually manipulated by the physical properties of the stimulus. For example, perceptual fluency was manipulated by font size (Rhodes and Castel, 2008), blurring (Yue et al., 2013), the contrast of target and background and different fonts (Magreehan et al., 2016), showing the text upright or inverted (Sungkhasettee et al., 2011), backward-mask (Besken and Mulligan, 2013), superimposing a checkerboard pattern mask on intact images (Besken, 2016), volume (Frank and Kuhlmann, 2017), replacing some portions of the speech signal with silence (Besken and Mulligan, 2014), etc. Usually, these manipulations are defaulted to be effective. However, few studies have measured the fluency of participants’ perceptual processing under different conditions to verify their manipulations of perceptual fluency are effective.

Previous researchers used self-paced study time and lexical decision time to measure perceptual fluency and found that some of the abovementioned manipulations of perceptual fluency were unsuccessful. For example, Mueller et al. (2014) found that font size did not affect self-paced study time and lexical decision time (also see Su et al., 2018). However, Yang et al. (2018) argued that it was inappropriate to measure perceptual fluency by self-paced study time and lexical decision time because self-paced study time and lexical decision time were too complex or affected by many other factors (Yap et al., 2015). Too much noise from other sources may overwhelm the difference in response time caused by perceptual characteristics. Yang et al. (2018) adopted a new paradigm – the continuous identification task to measure perceptual fluency. In our study, we also demonstrated that it was feasible to manipulate perceptual fluency by font size. Moreover, the continuous identification task was able to measure differences in perceptual fluency caused by font size successfully. Some other ways of manipulating perceptual fluency have not been measured. So, when using those ways to manipulate perceptual fluency, we need to measure the perceptual fluency to verify that the manipulation of perceptual fluency is effective.

### Limitations

Although font size affected JOLs (at least partially) through perceptual fluency, fluency explained only 6% in Experiment 1 and 18% in Experiment 4 of the font size effect on JOLs. This is inconsistent with the view of Koriat et al. (2004) that people making JOLs are based predominantly on fluency. This may be because the fluency commonly refers to the participant’s subjective feelings of the difficulty of information processing (Koriat, 1997; Oppenheimer, 2008). However, what we measured in this study was the objective fluency – response time. The objective response time and subjective feelings may not correspond exactly. Subsequent studies can try to measure perceptual fluency with subjective feelings.

## Conclusion

Perceptual fluency can affect JOLs in two ways: (1) in an analytical way through belief-based analysis and (2) in a non-analytical way.

## Data Availability Statement

The raw data supporting the conclusions of this article will be made available by the authors, without undue reservation.

## Ethics Statement

The studies involving human participants were reviewed and approved by The Ethics Committee of Northeast Normal University. The patients/participants provided their written informed consent to participate in this study.

## Author Contributions

ZW, CY, and YJ designed the experiment. ZW and WZ performed study. ZW and CY analyzed the data. ZW wrote the manuscript. All authors contributed to the article and approved the submitted version.

### Conflict of Interest

The authors declare that the research was conducted in the absence of any commercial or financial relationships that could be construed as a potential conflict of interest.
